# New insights into the role of mitochondrial calcium homeostasis in cell migration

**DOI:** 10.1016/j.bbrc.2017.05.039

**Published:** 2018-05-27

**Authors:** Vincent Paupe, Julien Prudent

**Affiliations:** Medical Research Council Mitochondrial Biology Unit, University of Cambridge, Wellcome Trust/MRC Building, Cambridge Biomedical Campus, Hills Road, Cambridge CB2 0XY, United Kingdom

**Keywords:** Mitochondria, Calcium, MCU, Cell migration

## Abstract

Mitochondria are dynamic organelles involved in numerous physiological functions. Beyond their function in ATP production, mitochondria regulate cell death, reactive oxygen species (ROS) generation, immunity and metabolism. Mitochondria also play a key role in the buffering of cytosolic calcium, and calcium transported into the matrix regulates mitochondrial metabolism. Recently, the identification of the mitochondrial calcium uniporter (MCU) and associated regulators has allowed the characterization of new physiological roles for calcium in both mitochondrial and cellular homeostasis. Indeed, recent work has highlighted the importance of mitochondrial calcium homeostasis in regulating cell migration. Cell migration is a property common to all metazoans and is critical to embryogenesis, cancer progression, wound-healing and immune surveillance. Previous work has established that cytoplasmic calcium is a key regulator of cell migration, as oscillations in cytosolic calcium activate cytoskeletal remodelling, actin contraction and focal adhesion (FA) turnover necessary for cell movement. Recent work using animal models and *in cellulo* experiments to genetically modulate MCU and partners have shed new light on the role of mitochondrial calcium dynamics in cytoskeletal remodelling through the modulation of ATP and ROS production, as well as intracellular calcium signalling. This review focuses on MCU and its regulators in cell migration during physiological and pathophysiological processes including development and cancer. We also present hypotheses to explain the molecular mechanisms by which MCU may regulate mitochondrial dynamics and motility to drive cell migration.

## Highlights

• Mitochondrial calcium dynamics control cell migration.
• MCU and regulators are involved in development and cancer invasion.
• MCU regulates cytoskeletal dynamics.
• MCUM modulates ATP and ROS production, and intracellular calcium signalling.

## 1 Introduction

Mitochondria are highly dynamic organelles that constantly undergo fusion and fission events to adapt their shape to the physiological needs of the cell. Mitochondrial plasticity allows their trafficking along the microtubules resulting in their strategic partitioning within the cell, which is crucial to ensure specialized functions such as immunity [1] and cell migration [2]. Mitochondria are also in dynamic contact with other organelles including the endoplasmic reticulum (ER). Transient contacts between ER and mitochondria are essential for a number of processes including autophagy, mitochondrial motility, lipid and calcium (Ca^2+^) fluxes and also mitochondrial division [3], [4]. The main actor of the mitochondrial division machinery is the large GTPase Dynamin-Related Protein 1 (Drp1) specifically recruited from the cytosol to ER-contact sites, where it oligomerizes and drives scission [5]. These contacts also allow Ca^2+^ transfer from ER to mitochondria enhancing the activity of tricarboxylic acid cycle (TCA) dehydrogenases required for oxidative phosphorylation [6]. Mitochondrial Ca^2+^ is also involved in the control of cell death [7] and reactive oxygen species (ROS) signalling [8]. It is now emerging that mitochondrial Ca^2+^ uptake also has a role in regulating cytosolic Ca^2+^ homeostasis and influences extracellular Ca^2+^ entry, which therefore might impact numerous cellular functions ranging from muscle contraction, neuron excitability and cell migration.

Cell migration is a natural process, essential for a number of physiological functions including embryonic development, immunity and wound-healing. This process is controlled by different regulatory effectors, which orchestrate the remodelling of the cytoskeleton architecture [9]. While the role of cytosolic Ca^2+^ in cell migration is well established, the function of mitochondrial Ca^2+^ and dynamics has only emerged recently. Indeed, many studies have shown that the mechanisms regulating cell migration are deregulated during metastasis, and Ca^2+^ signalling dysfunction is correlated with increased metastatic invasion and poor prognosis [10]. Thanks to the discovery of the mitochondrial Ca^2+^ uniporter (MCU) and its main regulators, the role of mitochondrial Ca^2+^ homeostasis in cell migration can be directly interrogated.

In this review, we will describe recent evidence highlighting the role of mitochondrial Ca^2+^ flux in cell migration. We will discuss the intimate connection between mitochondrial Ca^2+^ homeostasis and mitochondria dynamics/motility during this process.

### 1.1 The mitochondrial calcium homeostasis

**Fig. 1:**
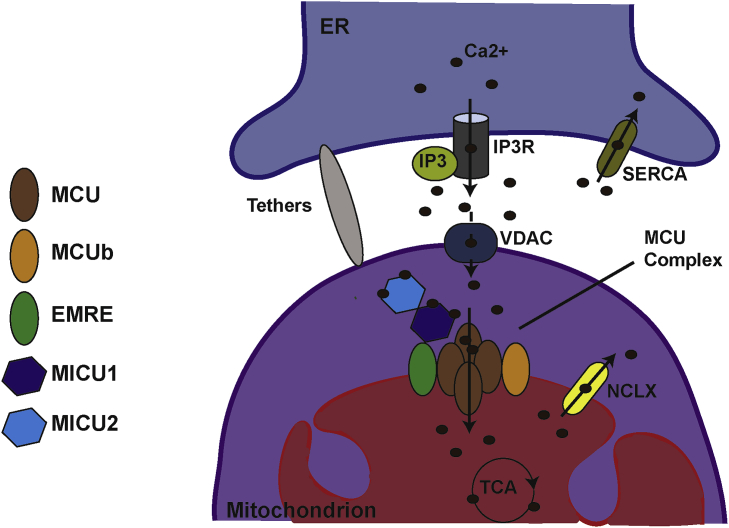
**Calcium homeostasis at the ER-mitochondria contact sites**. Under stimulation of the G protein-coupled receptor at the plasma membrane (PM), the phospholipase C (PLC) hydrolyses phosphatidylinositol 4,5-biphosphate (PIP2) into inositol 1,4,5-triphosphate inositol (IP3) and diacylgycerol (DAG). IP3 binds and activates the IP3 receptor (IP3R) leading to endoplasmic reticulum (ER) Ca^2+^ release in the cytosol or into neighbouring organelles. Due to the close proximity of the ER and the mitochondria, ensured by membrane tethering, highly localized and concentrated Ca^2+^ microdomains are specifically formed facilitating Ca^2+^ transfer to the mitochondria. Ca^2+^ first enters the mitochondria through the voltage-dependent anion channel (VDAC) at the OMM and then the mitochondrial calcium uniporter (MCU) transports it across the IMM. MCU is part of a complex, the MCU machinery (MCUM) composed of a negative regulator, MCUb, and EMRE, an essential IMM component required for the uniporter minimal activity. MCU is mainly regulated by members of the MICU family of proteins localized in the IMS, including MICU1 and MICU2 (because of the unknown function of MICU3 and its specific expression neuronal tissues, the latter is not represented in the model). MICU1 is considered as the MCU gatekeeper; at low cytosolic [Ca^2+^] MICU1 inhibits MCU activity whereas at high cytosolic [Ca^2+^], the binding of Ca^2+^ on MICU1 EF-hand leads to its conformational change and MCU channel activation. Ca^2+^ is extruded from the mitochondrial matrix by the IMM resident NCLX, which exchanges 1 Ca^2+^ for 3 Na^+^. The sarco/endoplasmic reticulum Ca^2+^ ATPase (SERCA) ensures the ER-refilling in Ca^2+^.

Intracellular Ca^2+^ signals are regulated by Ca^2+^ influx through the plasma membrane (PM) (extracellular [Ca^2+^] ≈ 1 mM) and Ca^2+^ release from intracellular stores, in particular from the Golgi ([Ca^2+^] ≈ 300 μM) and the ER ([Ca^2+^] ≈ 200–650 μM). To maintain the optimal cytosolic Ca^2+^ concentration (resting cytosolic [Ca^2+^] ≈ 100 nM), intracellular Ca^2+^ stores are constantly refilled while cytosolic Ca^2+^ is extruded from the cell by the plasma membrane Ca^2+^ ATPase (PMCA) pump. Intracellular Ca^2+^ is mainly stored in the ER lumen that is constantly refilled by the sarco/endoplasmic reticulum Ca^2+^-ATPase (SERCA) pump [11], [12]. Under stimulation, cell surface receptors activate the phospholipase C (PLC), which hydrolyses the membrane phospholipid phosphatidylinositol 4,5-bisphosphate (PIP2) to form inositol 1,4,5-trisphosphate (IP3) and diacylglycerol (DAG). IP3 diffuses then to the ER membrane, where it binds the IP3 receptor (IP3R), triggering Ca^2+^ release from the ER. ER Ca^2+^ can be released directly into the cytosol, or into juxtaposed organelles including mitochondria (Fig. 1) [13], the latter contributing to organelle Ca^2+^ homeostasis.

The affinity of MCU for Ca^2+^ is very low (Kd ≈ 10 μM), so the basal Ca^2+^ concentration in the cytosol is not sufficient to allow an efficient mitochondrial uptake [14]. Thus, large and localized Ca^2+^ concentrations are needed to activate MCU activity. It is now well established that one of the main functions of mitochondria-ER contact sites [15], stabilized by tethering proteins like mitofusin 2 (MFN2) [16], is to generate highly localized and concentrated Ca^2+^ microdomains facilitating Ca^2+^ transport into mitochondria [17]. When released from the ER, Ca^2+^ first passes the outer mitochondrial membrane (OMM) through the voltage-dependent anion channel (VDAC) [18] and then MCU [19], [20] transports it across the inner mitochondrial membrane (IMM) (Fig. 1). Mitochondrial Ca^2+^ uptake has been associated with energized mitochondria, where depletion of mitochondrial membrane potential abrogates mitochondrial Ca^2+^ uptake and defects in the respiratory chain have been associated with a decreased ability of mitochondria to pump Ca^2+^ [21].

Well before the discovery and the characterization of the uniporter, mitochondrial Ca^2+^ uptake has been associated with numerous physiological functions such as cell death, autophagy, skeletal muscle trophism, immunity, cardiomyocyte contraction and heart rate [22]. In the last six years, the discovery of MCU and it regulators have allowed us to reach a better understanding of the mitochondrial Ca^2+^ regulation and to investigate its role in intracellular Ca^2+^ signalling.

### 1.2 The mitochondrial calcium uptake machinery (MCUM)

After more than 50 years of intensive research, the composition of the uniporter has been finally resolved. It is composed of a pore-forming unit and regulatory subunits. The pore-forming subunit MCU was identified in 2011 by Mootha's and Rizzuto's groups [19], [20]. A number of studies confirmed the role of MCU in mitochondrial Ca^2+^ uptake in different animal species and cell types [22]. MCU is an evolutionarily conserved integral IMM protein harboring two transmembrane domains with the C- and N-terminus facing the matrix and a short loop in the IMS allowing Ca^2+^ entry [19]. Genomic analysis has identified a dominant negative form of MCU, MCUb, whose tissue expression profile differs from MCU [23]. Interestingly, reconstitution of the MCU complex in the yeast *Saccharomyces cerevisiae*, which lacks the mitochondrial uniporter, showed that expression of the human MCU alone was not sufficient to allow mitochondrial Ca^2+^ pumping activity [24]. However, co-expression with the IMM resident protein EMRE [25] was able to reconstitute the MCU channel activity in yeast, showing that EMRE is required to the minimal activity of the channel [24]. It has also been shown that EMRE was able to sense Ca^2+^ concentration through its C-terminus end, facing the mitochondrial matrix, and modulate MCU activity [26]. Blue native experiments have shown that MCU and EMRE assemble in high molecular weight complexes in association with different regulator subunits [23], [27], [28]. Among these regulators, one characterized even before the identification of MCU, was the mitochondrial Ca^2+^ uptake protein 1 (MICU1) [29], which belongs to the MICU family with MICU2 and MICU3. MICU1 is a soluble IMS protein, which can directly interact with MCU to modulate the channel activity depending on the cytosolic Ca^2+^ concentration. MICU1 is considered as the MCU “gatekeeper”. At low extramitochondrial Ca^2+^ concentration, MICU1 stabilizes MCU in a close state inhibiting Ca^2+^ uptake; whereas at high Ca^2+^ concentration, the EF-hand motif of MICU1 binds Ca^2+^ leading to MICU1 conformational change and subsequently the opening of the channel and Ca^2+^ entry [30], [31]. MICU2 has been shown to interact with MICU1 [32] and MICU3 [27] has been described to be specifically expressed in neuronal tissues. The specific role of these subunits remains to be clarified but this suggests additional complexity in the regulation of mitochondria Ca^2+^ entry that probably needs to be finely regulated in different tissues (see ([14]) for complete review).

### 1.3 Cytosolic calcium signalling during cell migration

**Fig. 2:**
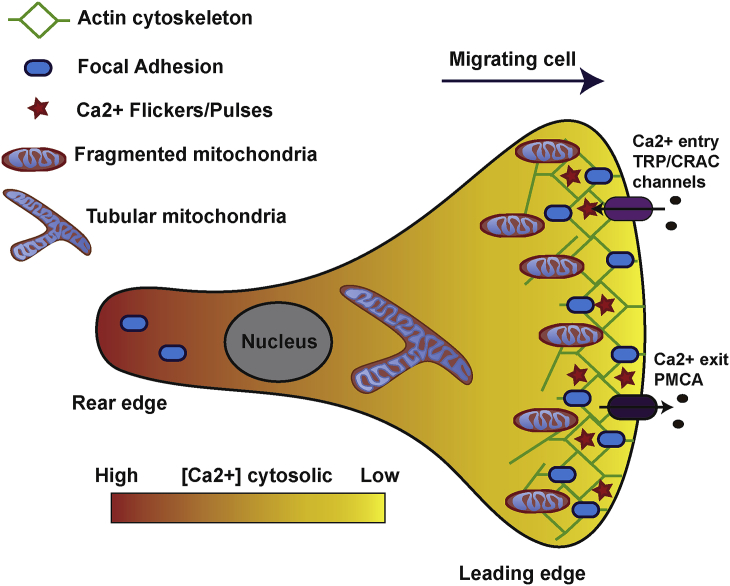
**Schematic representation of calcium signalling in a migrating cell**. During migration, cells exhibit a typical rear-to-front polarization. The cell migration machinery, including actin polarization and focal adhesion (FA) dynamics, is spatio-temporally regulated by cytosolic Ca^2+^. A [Ca^2+^] gradient is observed in the polarized cell, with a high [Ca^2+^] at the back required for calpain-dependent FA disassembly and with low [Ca^2+^] at the leading edge facilitating functional local Ca^2+^ pulses formation. This [Ca^2+^] gradient is ensured by the accumulation of the plasma membrane Ca^2+^ ATPase (PMCA) pump at the cell leading edge leading to Ca^2+^ extrusion. Local Ca^2+^ flickers/pulses at the leading edge are established by intracellular Ca^2+^ entry controlled by the transient receptor potential (TRP) channel or by a SOCE-STIM1/ORAI1-dependent mechanism. These localized Ca^2+^ microdomains allow actin-myosin contraction and FA assembly dynamics for cell migration. Mitochondrial drp1-dependent fission allows their relocalization to the leading edge, in order to generate ATP and ROS required for cytoskeleton remodelling. Mitochondria at the leading edge may also control the intracellular Ca^2+^ signalling, including store-operated calcium entry (SOCE) or ER Ca^2+^ release required for proper cell migration.

Directional cell migration involves three important steps: triggering and maintaining cell polarity, remodelling cytoskeleton to activate linear locomotion and modifying the direction of movement in response to gradients of environmental variations. Cell movement begins with the formation of protrusions of the cell membrane, followed by the establishment of new focal adhesions (FA) at the leading edge to anchor the cytoskeleton to the extracellular matrix (Fig. 2). Traction forces move the cell forward and the cycle ends with disassembly of the FA at the cell rear [9], [33]. Most of these events are spatio-temporally regulated by Ca^2+^ signalling [34]. Indeed, oscillations of cytosolic Ca^2+^ induce actin remodelling through the activation of the small GTPases RhoA and Rac1. During the rear-to-end retraction phase, actomyosin contraction is regulated by the phosphorylation of the myosin light chain (MLC) ensured by the calmodulin Ca^2+^-dependent kinase MLCK [10], [35]. Finally, the disassembly of the FA is controlled by the calpains, which are Ca^2+^-dependent proteases [36]. During migration, polarized cells exhibit a cytosolic Ca^2+^ gradient with low Ca^2+^ concentration at the leading edge [37] (Fig. 2). This gradient is ensured by an increased activity of the PMCA pumps at the leading edge to extrude intracellular Ca^2+^ [38]. This low Ca^2+^ concentration allows the different components of the cell migration machinery to respond to local pulses of intracellular Ca^2+^ changes. Recently, transient and localized microdomains of high Ca^2+^ concentrations have been shown to be more active at the front of the migrating cells (Fig. 2). These “Ca^2+^ flickers” or “pulses” have been shown to promote local focal adhesion proteins (FAP) disassembly [39] and steer the migrating cell in the direction of chemoatractants [40]. These hotspots of Ca^2+^ are dependent on the store-operated Ca^2+^ entry (SOCE) [38], [39], or on the activity of the stretch-activated receptor channel TRPM7 (transient receptor potential cation channel subfamily member 7) [40].

### 1.4 Store-operated calcium entry (SOCE) regulation

**Fig. 3:**
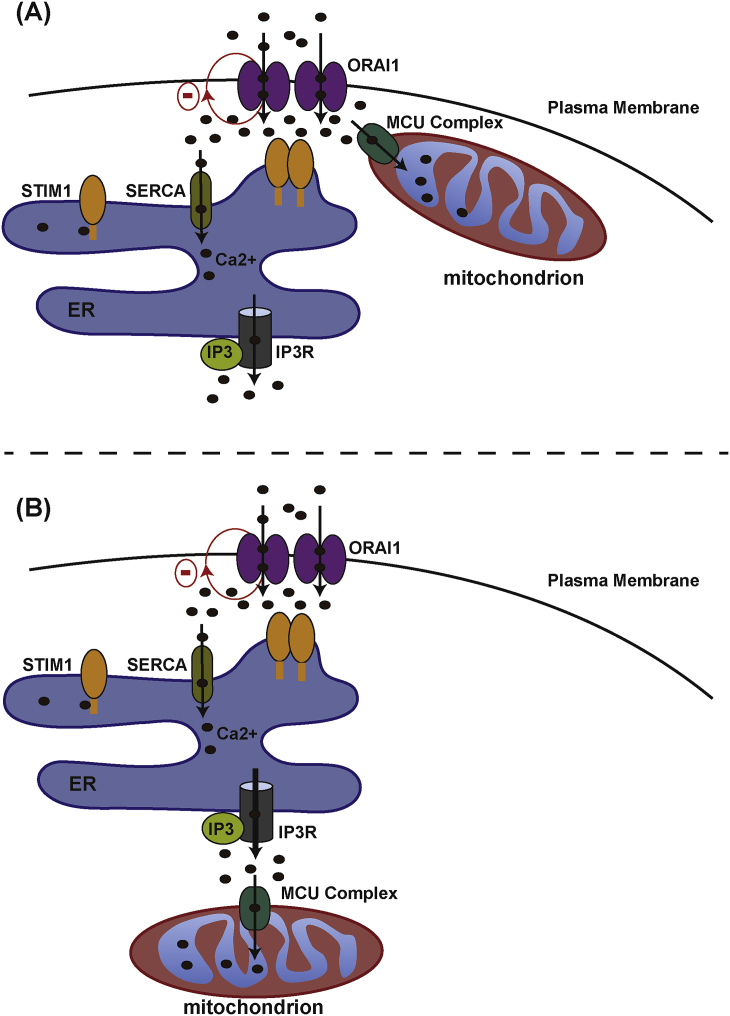
**Proposed models for the role of mitochondria in SOCE regulation**. SOCE is characterized by the extracellular Ca^2+^ entry controlled by ER Ca^2+^ store depletion. At low ER [Ca^2+^], Ca^2+^ dissociates form the ER-resident Stromal interacting molecule 1 (STIM1) allowing its oligomerization and relocalization at ER-PM contact sites. At these sites, STIM1 interacts with ORAI1 and activates the channel allowing Ca^2+^ entry. During this process, PCMA extrudes Ca^2+^ in the extracellular space and SERCA constantly refills the ER. In non-excitable cells, the contribution of mitochondria to SOCE regulation remains controversial. Mitochondria can be involved in SOCE regulation: **(A)** The immune cell model: During T-cell activation, mitochondria relocalize at the PM where they directly buffer Ca^2+^ entry. This reduces [Ca^2+^] at ER-PM contact sites and prevents the slow inactivation of ORAI1 by Ca^2+^ (Red circle arrow). (B) Alternative model: Due to steric hindrance at the ER-PM contact sites, mitochondria cannot directly buffer Ca^2+^ at these sites. Mitochondria contribute to ER Ca^2+^ store depletion by directly uptaking Ca^2+^ from the ER at the mitochondria-ER contact sites, contributing indirectly to SOCE activation.

The main path for Ca^2+^ entry in non-excitable cells is the SOCE allowing entry in the cell of extracellular Ca^2+^ through the PM-localized Ca^2+^-activated Ca^2+^-release channel ORAI1 [41]. The SOCE is mainly regulated by ER lumen Ca^2+^ concentration and the Ca^2+^ sensor ER-resident Stromal Interacting Molecule 1 (STIM1). Upon ER Ca^2+^-depletion, induced for example by sustained IP3 stimulation, cytosolic Ca^2+^ is extruded by PMCA and the ER refilled by SERCA. However, if the ER Ca^2+^ concentration remains too low, SOCE is activated by the STIM1/ORAI1 pathway in order to refill it [42] (Fig. 3). At low ER-Ca^2+^ concentration, Ca^2+^ dissociates from the STIM1-EF hand that senses the lumenal ER Ca^2+^, leading to the oligomerization of the protein. Cytoskeletal remodelling then promotes STIM1 relocalization specifically at ER-PM contact points where it interacts with ORAI1 and maintains the channel open [43] (Fig. 3). This regulation system allows a sustained phase of cytosolic Ca^2+^ influx required to maintain, for example, prolonged stimulation during cell migration.

Initial studies provided evidence that mitochondria can also play a role in SOCE regulation. Indeed, it has been shown that polarized and depolarized mitochondria led to an increase and decrease in the SOCE activity, respectively. The precise mechanism involved is still under debate, but some hypotheses point out a role of mitochondrial Ca^2+^ uptake. It has been proposed that mitochondria relocalize to the PM to directly buffer Ca^2+^ entry to inhibit the Ca^2+^-dependent inactivation of the ORAI1 channels (Fig. 3). Indeed, during T-cell activation mitochondria move toward the immune synapse and directly buffer SOCE induced Ca^2+^ entry [44] (Fig. 3A). In other models, an alternative mechanism proposed that mitochondria acts directly at the ER during IP3-induced Ca^2+^-release and helps buffer microdomains of Ca^2+^ [45] (Fig. 3B). It has been well documented that mitochondria can relocalize to the leading edge during cell migration [46], [47], [48] (Fig. 3) but their precise role in the process has remained elusive so far. We will present evidence that directly links the MCUM and some regulators of mitochondrial Ca^2+^ homeostasis to this process and review the possible associated mechanisms involving MCUM in intracellular Ca^2+^ and SOCE regulation, ATP and ROS production (Table 1).

**Table 1:** Effect of direct mitochondrial calcium uptake regulators on cell migration.

Gene	Relation to mitochondrial Ca^2+^ uptake	Genetic perturbation	Cell type/organism	Effect on cell migration	Direct mechanism proposed	Ref
MCU	Mitochondrial Ca^2+^ pore forming subunit	KO	- Mouse	Embryogenesis defect	–	[56], [58]
			- *C.elegans*	Wound healing defect	Decrease mtROS production required for Rho-1 inactivation	[95]
		KD	- Zebrafish	Cell migration and embryogenesis defects	Cytoskeleton and actin polymerization dynamics deregulation	[49]
			- TNBC	Inhibits cell migration and tumor growth	Decrease mtROS production and HIF1 signalling	[69]
			- MDA-MB-231	Delays in cell migration	SOCE inhibition	[129]
			- Hs578t	Decreases cell migration/cell polarization loss	Decrease of Actin/FAP dynamics, and Rho GTPases and calpain activities. Reduced SOCE activity	[70]
MICU1	Gatekeeper of MCU complex preventing mitochondrial calcium overload	KO	- Mouse	Perinatal lethality	–	[61], [62]
		KD	- hCVD-EC	Delay cell migration	–	[31]
			- Mouse-EC	Delay cell migration	–	[72]
Bcl-wav	Control mitochondrial calcium entry by interacting with VDAC	KD	- Zebrafish	Cell migration and embryogenesis defects	Actin dynamics defects	[49]
Bcl-xL	Control mitochondrial calcium entry by interacting with VDAC1/3	KD	- TNBC cells	Inhibition of cell migration	Inhibit mitochondrial Ca^2+^-induced ATP production.	[77]
		OE	- PanNET	Promotes cell migration and cell invasion *in vivo* in mouse	Increase cytoskeleton remodelling	[130]
Mcl-1	Promote mitochondrial calcium entry by interacting with VDAC1/3	OE	- NSCLC cells	Promote cell migration	Increase mtROS signalling	[87]
		KD	- NSCLC cells	Inhibition of cell migration	Decrease mtROS signalling	[87]

KO: Knock-out; KD: Knockdown; OE: overexpresson; TNBC: triple negative breast cancer; EC: endothelial cells; NSCLC: Non-small lung cancer cells.

## 2 MCUM and cell migration

### 2.1 MCUM deficiency *in vivo*

Increasing evidence support an active role for mitochondrial Ca^2+^ homeostasis on cell migration in different animal models. Genetic manipulation of the components of the MCUM, but also of direct or indirect regulators, have highlighted the contribution of mitochondrial Ca^2+^ in cell migration. In 2013, the first evidence for a role of the pore forming MCU in cell migration was provided by investigating its function in zebrafish early development [49]. Morpholino-dependent knockdown of MCU induced a dramatic decrease of the mitochondrial Ca^2+^ pool correlated with a marked increase of cytosolic Ca^2+^ level [49]. During zebrafish early development, and in particular during gastrulation, Ca^2+^-oscillations and Ca^2+^ waves play a crucial role in the cytoskeletal reorganization allowing guidance of the embryonic cells during migration [50], [51]. This disruption in intracellular Ca^2+^ signalling in morpholino-injected embyros (morphants) was associated with a deregulation of cell directionality and a decrease in actin polymerization dynamics leading to cell migration defects [49]. Other *in vivo* models have highlighted physiological functions of MCU. An elegant study showed that loss of the nematode orthologue of MCU (MCU-1) suppressed mitochondrial Ca^2+^ uptake and impaired wound healing [52]. The authors show, using cytosolic and mitochondrial targeted Ca^2+^ sensitive GCaMP3 fluorescent probes, that a mitochondrial Ca^2+^ wave, induced by the cytosolic Ca^2+^ wave occurs after wounding. This wave of mitochondrial Ca^2+^ was totally inhibited in MCU-1 knockout preventing cytoskeleton remodelling during the healing process [52]. Despite the difference between epidermal structures among organisms, some key features of wound-healing seem to be conserved between vertebrates and invertebrates [53]. An almost universal signal triggered by wounding is an elevation of intracellular Ca^2+^ at wound sites to locally recruit polymerized actin. In fact, it was described that wounding induced Ca^2+^ waves in epithelial cells that were crucial to increase cell motility rate [54], [55]. These data obtained in the zebrafish and the nematode emphasize the role of MCU in Ca^2+^ signalling linked to the regulation of cytoskeleton remodelling.

Surprisingly, the total MCU-KO in a mixed genetic mice background (outbred CD1 strain) exhibits only a discrete phenotype with a reduced exercise tolerance and skeletal muscle respiration correlating to a defect in PDH phosphorylation [56]. The role of MCU in cellular bioenergetics has also been shown in the control of the response of the B-adrenergic stimuli on heart rate [57]. The absence of phenotype in mouse embryogenesis was quite unexpected. Although the mice were significantly smaller, development seemed to happen normally. However, MCU-KO was embryonic lethal in the inbred C57BL/6 mice background and the outbred CD1 mice did not follow a mendelian transmission suggesting early defects during embryogenesis [56], [58]. These results also point out the possibility of an unknown compensatory mechanism allowing adaptation of some mouse embryonic cells [59] or the existence of a sufficient MCU-independent Ca^2+^ entry [60] during development in mammals. Interestingly, two groups have recently characterized the MICU1-KO mouse with different phenotypes [61], [62]. Both groups reported an increase in the resting mitochondrial Ca^2+^ level and a decreased capacity for mitochondria to uptake Ca^2+^ at high concentration (>15 μM). However, one study showed that MICU1-KO in C57BL/6 J background was lethal a few hours after birth due to failure in basic vital functions [61], whereas the other obtained a high perinatal mortality in C57BL/6 N KO mice [62]. Surviving mice exhibited neurological and myopathic defects similar to the symptoms observed in patients harboring MICU1 mutations [63], [64], [65], however these defects improved with time, highlighting again the existence of a potential compensatory mechanism.

Taken together, these studies indicate that deregulation of mitochondrial Ca^2+^ homeostasis can lead to an alteration of cell migration via defects in actin dynamics or premature embryonic death.

### 2.2 Effect of MCUM deficiency in cell migration

The regulation of cell migration plays a major role in tumor metastasis allowing the movement of cancer cells to the periphery and the circulation. In prostate and colon cancers, it has been shown that overexpressed microRNA specifically downregulating MCU and dampening mitochondrial Ca^2+^ uptake resulted in enhanced cell resistance to apoptosis [66]. On another other hand, MCU and mitochondrial Ca^2+^ up-regulation can greatly enhance metastatic behavior. Recently, clinical data analysis of breast cancer patients has associated overexpression of MCU and downregulation of MICU1 to poor prognosis [67], [68] suggesting that mitochondrial Ca^2+^ uptake accelerates cancer dissemination. Indeed, multiple *in vitro* studies using triple negative breast cancer (TNBC) and other breast cancer cell models have shown that depletion of MCU led to a drastic cell migration decrease, independent of cell proliferation [67], [69], [70]. These defects were characterized by a delay in gap closure after scratch assay and/or a decrease in the number of migrating cells in Boyden chamber analysis. Moreover, silencing MCU in TNBC cells strikingly inhibited *in vivo* tumor growth and metastasis progression in mice [69]. Specific inhibition of mitochondrial Ca^2+^ uptake by the Ruthenium 360, a potent inhibitor of MCU, also led to decreased cell migration capacity [67], [70]. Interestingly, stable knockdown of MCU in Hs578t cells led to an increase in actin stiffness, loss of cell polarization and an impairment of the FAP dynamics [70]. In MCU-silenced cells, the polarity of the cells during migration was lost due to a decrease in the activation of the Rho GTPases, RhoA and Rac1 activities, analyzed by FRET and pull down experiments. Moreover, cytoskeletal dynamics via phosphorylation of MLC was downregulated, and the turnover of the FAP, Vinculin and Paxillin, was delayed due to the decreased activity of the calpains proteases [70]. These effects were attributed to a decrease of intracellular Ca^2+^ signalling from the ER and cytosol.

It is now generally accepted that MICU1 serves as a molecular gatekeeper preventing mitochondria Ca^2+^ overload (for review see [71]). Knockdown of MICU1 in endothelial cells (EC) impaired cell migration in scratch assays [31]. Cultured human EC, derived from cardiovascular disease patients (CVD-EC), showed a marked decrease in mRNA for MICU1, but not MCU relative to healthy control EC [72]. This led to a basal increase of mitochondrial Ca^2+^ and migration deficiency. Re-expression of MICU1 in those cells reduced the mitochondrial Ca^2+^ accumulation, which correlated with CVD-EC ability to increase cell migration [72]. Now, it is questionable why MICU1 loss does not facilitate cell migration as it actually increases mitochondrial Ca^2+^ content. Interestingly, it has been reported that silencing MICU1 increases mitochondrial Ca^2+^ uptake at low cytosolic Ca^2+^ concentration but also strongly inhibits mitochondrial Ca^2+^ uptake in response to agonist-induced Ca^2+^ rises [30]. Thus, MICU1 deficient mitochondria would fail to relay and buffer cytosolic Ca^2+^ waves triggered during the cell migration process (this will be discussed later in the review).

Evidence is therefore emerging that mitochondrial Ca^2+^ dynamics are important in cell migration. The challenge is to determine whether these roles are direct and/or indirect, and identify the molecular mechanisms that couple these dynamics to the signalling pathways that drive the migration process.

## 3 Mechanisms by which MCUM deficiency alters cell migration

### 3.1 Role of MCUM in ATP production

Cytoskeleton dynamics is an active process that is directly dependent on ATP levels. Given the regulatory role of mitochondrial Ca^2+^ on TCA enzyme activities, a deficiency of MCUM could potentially alter mitochondrial ATP production and subsequently global or local cytoskeleton remodelling. It has been put forward that a deficiency of MCU affects ATP production by decreasing resting mitochondrial Ca^2+^ levels. However, numerous studies reported that different cell lines with MCU deficiency did not exhibit respiration defect in basal conditions. Indeed, no detectable difference in oxygen consumption was noted in MCU-KO derived fibroblasts [56], and neither in MCU-silenced HeLa cells under basal conditions [19]. Thus MCU silencing appears to have surprisingly little impact on mitochondrial bioenergetics in non-excitable cells [20], [73]. Of note, modulation of MCU levels did not affect the ATP content in rat neonatal cardiomyocytes either [74]. Nevertheless, it seems that the effect of MCU loss on ATP production could be significant in tissues that have a high-energy demand as in the skeletal muscle of MCU-KO mouse, which exhibited alterations in the phosphorylation and activity of pyruvate dehydrogenase [56], and also in pancreatic β-cells where glucose-stimulated ATP increases, necessary for triggering insulin exocytosis, required a functional MCU [75], [76]. Moreover, in MCU-deficient TNBC cells, inhibiting glycolysis by 2-deoxy-d-glucose treatment failed to induce an increase of ATP production [69]. This illustrates that upon an increase of energy demand, non-excitable cells lacking MCU can also exhibit a global ATP production defect. In Hs578t breast cancer cells, the authors reported no alteration in the total level of intracellular ATP associated to cytoskeleton defects upon MCU loss [70]. However, even if the global intracellular ATP production is not decreased, a decreased capacity to produce rapid, localized boosts of ATP could alter local actin cytoskeleton remodelling, and myosin-dependent contraction. In TNBC cells, Bcl-xL silencing affected the mitochondrial Ca^2+^-induced ATP raise, stimulated by the cytokine cl-CD95L and impaired cell migration [77] but so far, no study has confirmed that this ATP defect is directly responsible for cytoskeleton remodelling defects during cell migration.

In contrast to the loss of mitochondrial Ca^2+^ in MCU deficient cells, the MICU1 deficiency that resulted in migration defects was associated with a consistent increase of resting mitochondrial Ca^2+^ levels. This rise in mitochondrial Ca^2+^ has been correlated to a better response to increased ATP demand [78]. Since MCU and MICU1 deficiency show similar issues in cell migration with opposing matrix Ca^2+^ load, variation in ATP levels is not likely to play a major role in the migratory phenotypes of MCUM deficient cells; rather the dynamics of the Ca^2+^ wave regulation may be more crucial.

### 3.2 Role of MCUM in ROS signalling

In 2016, Rizzuto's group proposed a mechanism involving mitochondrial MCU-regulated ROS production during cell migration in triple negative breast cancer (TNBC) cell lines [69]. MCU was silenced in three TNBC cell lines, which strikingly inhibited cell migration, *in vivo* tumor growth and metastasis progression [69]. Excessive ROS levels are toxic but sub-lethal production contributes to important signalling functions, particularly in cancers in which it has been shown that ROS promote cell proliferation, migration and invasion [79]. Loss of MCU resulted in the inhibition of mitochondrial ROS (mtROS) production, which led to a reduction in the expression of the hypoxia induced factor 1a (HIF1α) transcription factor. This defect resulted in failure to activate the hypoxic program essential for cell invasion *in vitro* and *in vivo* [69]. These data reinforced previous evidence linking spikes in mitochondrial Ca^2+^ concentration with increased mtROS production [80].

Links between mitochondrial Ca^2+^, ROS and cell migration have also been made with the Bcl-2 family of proteins. Beyond their role as key modulators of apoptosis [81], these multifactor proteins also participate in multiple functions including cell migration [82], [83], [84]. In the zebrafish model, loss of the pro-apoptotic protein Bcl-wav led to acute defects in cell migration during embryogenesis due to defects in actin dynamics remodelling. This was directly attributed to Bcl-wav ability to interact with VDAC1 and promote mitochondrial Ca^2+^ uptake [49], [59]. Other members of the Bcl-2 family, including Bcl-xL and Mcl-1, have been shown to regulate mitochondrial Ca^2+^ homeostasis via their direct interaction with VDAC1/3 [77], [85], [86], [87] and promote cell migration [77], [87]. Moreover, by studying the effect of an apoptosis defective mutant of Bcl-xL in cancer cell lines, Soyoung et al. show that Bcl-xL promoted cell migration and metastasis in mice independently from its anti-apoptotic function [88]. Similarly, high Mcl-1 expression promoted cell migration but not proliferation in non-small cell lung cancer (NSCLC) cells and knockdown of Mcl-1 in this cell line inhibited cell migration in scratch wound-healing assays [87]. Interestingly, the migratory delay in Mcl-1 knockdown cells was associated with a decrease in mtROS production, which was rescued upon restoration of ROS levels [87]. These data are consistent with the idea that ROS production can drive migration in lung cancer by a mechanism involving Ca^2+^ channeling through VDAC. The exact mechanism of ROS action is still controversial, however pioneering work showed that ROS produced within migrating cells promotes cell movement and are necessary for chemotaxis [89]. If generation of anion superoxide might act as a redox signal itself, it is rapidly degraded by superoxide dismutase (SOD) to hydrogen peroxide (H_2_0_2_), which is believed to induce cellular changes by reversibly oxidizing the thiol group of cysteine residues of specific proteins [90]. H_2_0_2_ levels are regulated by peroxiredoxins, which have been shown to regulate ROS-mediated signal transduction in mammalian cells; for example, peroxiredoxin-2 overexpression has been shown to suppress chemotactic migration and adhesion induced by platelet-derived growth factor (PDGF) in MEFs [91]. Moreover, ablation of the protein *in vivo* promoted the growth and migration of smooth muscle cells during vascular remodelling [91]. However, the oxidized proteins that modulate migration and adhesion are unknown in most instances. Adhesion dynamics drive the migration cycle by activating Rho GTPases, which in turn regulate actin polymerization and myosin II activity [92]. In HeLa cells, the mechanism by which ROS facilitate cell spreading involves downregulation of RhoA activity through activation of p190Rho-GAP, a negative regulator of RhoA [93]. In *C.* *elegans*, it has been shown that wound closure by F-actin accumulation at wound site, requires the Cdc42 small GTPase and Arp2/3-dependent actin polymerization and is negatively regulated by RHO-1 and non-muscle myosin [94]. In the *c.* *elegans* model of MCU-1 KO leading to wound-healing defects, Xu et al. demonstrated that mitochondrial Ca^2+^ was totally inhibited in MCU-1 knockout and the mtROS production necessary to remodel the cytoskeleton during healing was prevented. The authors proposed that mtROS inhibited RHO-1 activity by oxidizing its redox sensitive motif to promote wound closure [95].

Collectively, these studies show that mtROS production are involved in cell migration and their release in the cytoplasm requires a functional MCU. However, in MICU1 deficient EDV cells where cell migration was decreased, the authors observed a large increase of mtROS [31], [72]. Therefore it is likely not the mtROS pool itself, but rather its local release, which is important for cell migration. Although it is still not clear how mitochondrial Ca^2+^ regulates this release, Booth et al. recently demonstrated the interdependence of mitochondrial Ca^2+^ uptake and H_2_0_2_ release at the mitochondria-ER interface [96]. The authors suggested that nanodomains of H_2_O_2_ accumulating in the mitochondrial cristae are compressed and released at mitochondria-ER contact sites upon Ca^2+^ signal propagation to the mitochondria, likely due to concomitant K+ and water influx to the matrix. Transient release of H_2_O_2_ in turn, would sensitize ER Ca^2+^ release to maintain Ca^2+^ oscillations [96]. It seems that the capacity of mitochondria to uptake large amount of Ca^2+^ at membrane contact sites is crucial for the generation and release of mtROS required for proper cell migration.

### 3.3 Role of MCUM in SOCE regulation

Mitochondria are scattered throughout the cytoplasm but their distribution can vary depending on local high-energy demands. For example, human ovarian adenocarcinoma cells increase local AMP-activated protein kinase (AMPK) activated mitochondria in cellular protrusions to respond to metabolic demands [97]. During cancer cell migration, actin polymerization, lamelipodia formation and FAP dynamics occur at the leading edge of the cell and require high-energy production and Ca^2+^ buffering [37]. Mitochondrial relocalization at these sites has been shown to be critical to ensure proper cell migration and raises the question of their function at these specific sites. Beside their role in ATP and ROS production, a non-exclusive hypothesis is that mitochondria are important to buffer high Ca^2+^ pulses but also to directly control local extracellular Ca^2+^ entry at the PM during SOCE. Early work has provided solid evidence demonstrating that mitochondria can play a role in controlling the opening of the Ca^2+^ release-activated channels (CRAC) that regulate cellular Ca^2+^ entry through SOCE regulation [98], [99], [100], [101], [102], [103] (Fig. 3). This function in immune cells, and in particular during T-cell activation, has been well described [98], [104], [105], [106]. However, in other non-excitable cell types the precise function of mitochondria needs further analysis (Fig. 3). Genetic manipulation of MCU has revealed the requirement for mitochondrial Ca^2+^ homeostasis in regulating transient fluxes of cytosolic Ca^2+^. For example, it has been shown that MCU regulated leukotriene-induced physiological oscillations of cytoplasmic Ca^2+^ in a rat basophile cell line (RBL-1) [107]. The authors showed that pro-inflammatory leukotriene-induced cytoplasmic oscillations triggered similar (in number and frequency) oscillations of mitochondrial Ca^2+^, which were inhibited by mitochondrial membrane depolarisation or MCU knockdown. Moreover knockdown of MCU induced a reduction in cytosolic Ca^2+^ oscillations indicating a proactive role of mitochondria in the propagation of physiological cytosolic Ca^2+^ signalling [107]. Other studies in breast cancer [67], [70] and neuroblastoma cells [108] have also highlighted a SOCE defect when MCU was silenced, and the requirement for mitochondrial Ca^2+^ uptake to ensure IP3-induced STIM1 oligomerization in HeLa cells [109].

For now, only a few studies have investigated the role of the uniporter on SOCE during cell migration, and this idea has been challenged by contradictory results. The role of SOCE regulated by STIM1 has been shown to control the actomyosin contractility during breast cancer cell migration [110]. STIM1 silencing inhibited the recruitment and association of active FA kinase and talin at FA, and prevented myosin II phosphorylation necessary for contractility [111]. Interestingly, two independent studies, in MDA-MB-231 [67] and Hs578t [70] breast cancer cell lines have associated MCU loss to a SOCE defect where MCU silencing considerably reduced cytosolic Ca^2+^ entry after ER-store depletion upon thapsigargin treatment. In these studies, inhibition of the SOCE by pharmaceutical approaches [67] or STIM1 silencing [70], phenocopies cell migration and actin/FAP dynamics defects induced by *mcu*-loss. These data suggest that mitochondrial Ca^2+^ homeostasis may control cytoskeleton dynamics and cell migration via the SOCE regulation. On the other hand, it has been reported that MCU silencing did not affect SOCE in three different breast cancer cell lines, attributing cell migration defects to a decrease in ROS production and HIF1 signalling [69]. These data corroborate other studies stating that mitochondria are not directly involved in the buffering of Ca^2+^ entry during SOCE [112]. Because of the fast Ca^2+^-dependent inactivation of the CRAC channels, mitochondria would need to be closely located to the PM to influence this process (d < 30 nm). It has been shown that in contrast to ER Ca^2+^ release, Ca^2+^ entry via SOCE did not generate Ca^2+^ hotspots at the OMM. This suggests that mitochondria could not relocalize at ER-PM contact, where SOCE occurs, due to steric hindrance [113]. However, it can be hypothesized than mitochondria can buffer Ca^2+^ generated during SOCE by acting directly at the ER (Fig. 3). Thus, mitochondrial Ca^2+^ uptake could participate indirectly in STIM1-regulated SOCE activation by buffering ER Ca^2+^ release. It should be noted that the discrepancy between these different studies might depend on the cell types and agonists used to activate SOCE. Thus, further studies will be needed to fully assess this question.

Recently, in human lung fibroblasts and umbilical vein EC (HUVEC), local high Ca^2+^ microdomains (Ca^2+^ flickers or pulses) have been identified and shown to be most active at the leading edge of migrating cells [38], [39], [40] (Fig. 2). When cells are exposed to a growth factor gradient perpendicular to cell movement, asymmetric TRP-dependent Ca^2+^ flicker activity develops across the lamella and promotes the turning of the cells towards the chemo-attractant [40]. Moreover, Tsai et al. showed that Ca^2+^ pulses restricted to the leading edge were generated by IP3-stimulated local depletion of ER Ca^2+^, and local activation of STIM1, supporting pulsatile front retraction and adhesion of the cell [38], [39]. They also have been shown to control local MLCK activation and FA dynamics. So far, no studies have been performed to elucidate the potential role of MCU and the Ca^2+^ buffer capacity of the mitochondria in this phenomenon. It is tempting to hypothesize that MCU may also regulate the intensity and the duration of those flickers. MCU may act on these Ca^2+^ flickers in two different ways: by its potential ability to regulate SOCE, or by direct Ca^2+^ buffering at the ER. Therefore, it will be important to design new experiments in migrating cells to elucidate the role of MCUM in this phenomenon.

### 3.4 Potential role of MCUM in mitochondrial motility and dynamics

As described previously, mitochondria need to relocalize to the leading edge of the migrating cells to ensure their function (Fig. 2). Mitochondrial Ca^2+^ uptake may also impact cell migration via its capacity to regulate this process. So far, the role of MCU in mitochondrial motility and dynamics has not been fully investigated. Mitochondrial motility along microtubules is ensured by mitochondrial adaptor, motor proteins and cytoskeleton components, which are regulated by cytosolic Ca^2+^ [114]. The GTPase mitochondrial protein Miro1 contains two EF-hand Ca^2+^ binding domains and plays an important role in mitochondrial motility along microtubules. High cytoplasmic Ca^2+^ levels halt mitochondrial movement by binding to miro1 EF-hand domains [115], [116]. In 2011, Chang et al. demonstrated that intra-mitochondrial Ca^2+^ can also play a critical role in mitochondrial transport along the axons [117]. The authors showed that mitochondrial Ca^2+^ content was inversely proportional to the speed of mitochondrial movement. Thus, by regulating intracellular Ca^2+^ signal, MCU may indirectly control mitochondrial motility.

Mitochondrial motility is also regulated by mitochondrial shape and it has been shown that fragmented and smaller mitochondria move faster along microtubules. Increasing evidence in multiple studies has shown that mitochondrial dynamics are involved in cell migration and cancer invasiveness [46], [48], [118]. Indeed, mitochondrial fragmentation is required for cancer cell migration and invasion [119], [120] as Drp1 phosphorylation at Ser616 allows mitochondrial trafficking to the leading edge [121]. Drp1 recruitment to mitochondria is regulated by the phosphatase calcineurin dephosphorylating Drp1 at Ser637 upon a rise of cytosolic Ca^2+^ [122], [123]. Thus by modulating cytosolic Ca^2+^ levels, MCUM is likely to affect mitochondrial dynamics and distribution, which may impact cell migration. In mouse embryonic fibroblasts derived from MCU-KO, the mitochondrial shape was not altered by MCU loss [56]. However, mitochondria from MICU1 patients with high mitochondrial Ca^2+^ pool harbored fragmented mitochondria [63]. This was consistent with previous work showing that thapsigargin treatment on cultured cells induced Ca^2+^ influx into mitochondria that drove mitochondrial fragmentation [124]. Additionally, in two models of ischemia/reperfusion injury, mitochondrial Ca^2+^ was required for mitochondrial fragmentation by modulating Drp1 level [125], [126]. Finally, a recent study highlighted the role for MCU in mitochondrial fragmentation via accumulation of Drp1 Ser616 required for neutrophil polarization and chemotaxis [127]. Those studies support the idea of a role of mitochondrial Ca^2+^ uptake in mitochondrial dynamics.

Thus, it will be interesting to further investigate the specific role of MCUM in mitochondrial motility/dynamics during cell migration.

## 4 Conclusions

Recent evidence has shed a light on the crucial role of MCU and its regulators in cell migration. Independently of the role of MCU in cancer cell migration, different studies have highlighted the role of mitochondrial Ca^2+^ homeostasis in immune cell polarization and chemotaxis [127], [128]. Taken together, these data obtained in different specialized cells and animal models highlight the crucial and evolutionarily conserved function of MCU in cell migration from worms to vertebrates.

If MCU is involved in cytoskeleton remodelling, the full mechanism of its impact on cell migration remains to be discovered. So far, the non-exclusive connections between ATP and ROS production and cytosolic Ca^2+^ signal regulation have been investigated. To fully elucidate these mechanisms, it will be important to decipher the potential role of the MCUM on Ca^2+^ flickers formation/intensity but also the impact of MCUM on mitochondrial dynamics at the leading edge during cell migration.

Finally, the fact that MCU is overexpressed in breast cancer patients and the clear evidence linking MCU to cancer invasion and growth, points to mitochondrial Ca^2+^ uptake as a potential therapeutic target in highly proliferative cancers.

## References

[1] Martín-Cófreces N.B., Baixauli F., Sánchez-Madrid F. (2014). Immune synapse: conductor of orchestrated organelle movement. Trends Cell Biol..

[2] Da Silva A.F., Mariotti F.R., Máximo V., Campello S. (2014). Mitochondria dynamism: of shape, transport and cell migration. Cell Mol. Life Sci..

[3] Phillips M.J., Voeltz G.K. (2015). Structure and function of ER membrane contact sites with other organelles. Nat. Rev. Mol. Cell Biol..

[4] Naon D., Scorrano L. (2014). At the right distance: ER-mitochondria juxtaposition in cell life and death. Biochim. Biophys. Acta.

[5] Friedman J.R., Lackner L.L., West M., DiBenedetto J.R., Nunnari J., Voeltz G.K. (2011). ER tubules mark sites of mitochondrial division. Science.

[6] Murgia M., Rizzuto R. (2015). Molecular diversity and pleiotropic role of the mitochondrial calcium uniporter. Cell Calcium.

[7] Prudent J., McBride H.M. (2017). The mitochondria–endoplasmic reticulum contact sites: a signalling platform for cell death. Curr. Opin. Cell Biol..

[8] Hempel N., Trebak M. (2017). Crosstalk between calcium and reactive oxygen species signaling in cancer. Cell Calcium.

[9] Gardel M.L., Schneider I.C., Aratyn-Schaus Y., Waterman C.M. (2010). Mechanical integration of actin and adhesion dynamics in cell migration. Annu. Rev. Cell Dev. Biol..

[10] Chen Y.-F., Chen Y.-T., Chiu W.-T., Shen M.-R. (2013). Remodeling of calcium signaling in tumor progression. J. Biomed. Sci..

[11] Raffaello A., Mammucari C., Gherardi G., Rizzuto R. (2016). Calcium at the center of cell signaling: interplay between endoplasmic reticulum, mitochondria, and lysosomes. Trends biochem. Sci..

[12] Berridge M.J., Lipp P., Bootman M.D. (2000). The versitality and universality of calcium signalling. Nat. Rev. Mol. Cell Biol..

[13] Rizzuto R. (1998). Close contacts with the endoplasmic reticulum as determinants of mitochondrial Ca2+ responses. Science.

[14] De Stefani D., Rizzuto R., Pozzan T. (2016). Enjoy the trip: calcium in mitochondria back and forth. Annu. Rev. Biochem..

[15] Giacomello M., Pellegrini L. (2016). The coming of age of the mitochondria–ER contact: a matter of thickness. Cell Death Differ..

[16] de Brito O.M., Scorrano L. (2008). Mitofusin 2 tethers endoplasmic reticulum to mitochondria. Nature.

[17] Rizzuto R., Brini M., Murgia M., Pozzan T. (1993). Microdomains with high Ca2+ close to IP3-sensitive channels that are sensed by neighboring mitochondria. Science.

[18] De Stefani D., Bononi a, Romagnoli a, Messina a, De Pinto V., Pinton P., Rizzuto R. (2012). VDAC1 selectively transfers apoptotic Ca2+ signals to mitochondria. Cell Death Differ..

[19] Baughman J.M., Perocchi F., Girgis H.S., Plovanich M., Belcher-Timme C.A., Sancak Y., Bao X.R., Strittmatter L., Goldberger O., Bogorad R.L., Koteliansky V., Mootha V.K. (2011). Integrative genomics identifies MCU as an essential component of the mitochondrial calcium uniporter. Nature.

[20] De Stefani D., Raffaello A., Teardo E., Szabò I., Rizzuto R. (2011). A forty-kilodalton protein of the inner membrane is the mitochondrial calcium uniporter. Nature.

[21] Paupe V., Prudent J., Dassa E.P., Rendon O.Z., Shoubridge E.A. (2015). CCDC90A (MCUR1) is a cytochrome c oxidase assembly factor and not a regulator of the mitochondrial calcium uniporter. Cell Metab..

[22] Kamer K.J., Mootha V.K. (2015). The molecular era of the mitochondrial calcium uniporter. Nat. Rev. Mol. Cell Biol..

[23] Raffaello A., De Stefani D., Sabbadin D., Teardo E., Merli G., Picard A., Checchetto V., Moro S., Szabò I., Rizzuto R. (2013). The mitochondrial calcium uniporter is a multimer that can include a dominant-negative pore-forming subunit. EMBO J..

[24] Kovács-bogdán E., Sancak Y., Kamer K.J., Plovanich M., Jambhekar A. (2014). Reconstitution of the Mitochondrial Calcium Uniporter in Yeast.

[25] Sancak Y., Markhard A.L., Kitami T., Kovács-Bogdán E., Kamer K.J., Udeshi N.D., Carr S.a, Chaudhuri D., Clapham D.E., Li A.a, Calvo S.E., Goldberger O., Mootha V.K. (2013). EMRE is an essential component of the mitochondrial calcium uniporter complex. Science.

[26] Vais H., Mallilankaraman K., Mak D.O.D., Hoff H., Payne R., Tanis J.E., Foskett J.K. (2016). EMRE is a matrix Ca2+ sensor that governs gatekeeping of the mitochondrial Ca2+ uniporter. Cell Rep..

[27] Plovanich M., Bogorad R.L., Sancak Y., Kamer K.J., Strittmatter L., Li A.A., Girgis H.S., Kuchimanchi S., De Groot J., Speciner L., Taneja N., Oshea J., Koteliansky V., Mootha V.K. (2013). MICU2, a paralog of MICU1, resides within the mitochondrial uniporter complex to regulate calcium handling. PLoS One.

[28] König T., Tröder S.E., Bakka K., Korwitz A., Richter-Dennerlein R., Lampe P.A.A., Patron M., Mühlmeister M., Guerrero-Castillo S., Brandt U., Decker T., Lauria I., Paggio A., Rizzuto R., Rugarli E.I.I., De Stefani D., Langer T., König T., Tröder S.E., Bakka K., Korwitz A., Richter-Dennerlein R., Lampe P.A.A., Patron M., Mühlmeister M., Guerrero-Castillo S., Brandt U., Decker T., Lauria I., Paggio A., Rizzuto R., Rugarli E.I.I., De Stefani D., Langer T. (2016). The m-AAA protease associated with neurodegeneration limits MCU activity in mitochondria. Mol. Cell.

[29] Perocchi F., Gohil V.M., Girgis H.S., Bao X.R., McCombs J.E., Palmer A.E., Mootha V.K. (2010). MICU1 encodes a mitochondrial EF hand protein required for Ca(2+) uptake. Nature.

[30] Csordás G., Golenár T., Seifert E.L., Kamer K.J., Sancak Y., Perocchi F., Moffat C., Weaver D., Perez S.D.L.F., Bogorad R., Koteliansky V., Adijanto J., Mootha V.K., Hajnóczky G. (2013). MICU1 controls both the threshold and cooperative activation of the mitochondrial Ca2+ uniporter. Cell Metab..

[31] Mallilankaraman K., Doonan P., Cárdenas C., Chandramoorthy H.C., Müller M., Miller R., Hoffman N.E., Gandhirajan R.K., Molgó J., Birnbaum M.J., Rothberg B.S., Mak D.-O.D., Foskett J.K., Madesh M. (2012). MICU1 is an essential gatekeeper for MCU-mediated mitochondrial Ca(2+) uptake that regulates cell survival. Cell.

[32] Patron M., Checchetto V., Raffaello A., Teardo E., VecellioReane D., Mantoan M., Granatiero V., Szabò I., DeStefani D., Rizzuto R. (2014). MICU1 and MICU2 finely tune the mitochondrial Ca2+ uniporter by exerting opposite effects on MCU activity. Mol. Cell.

[33] Parsons J.T., Horwitz A.R., Schwartz M.A. (2010). Cell adhesion: integrating cytoskeletal dynamics and cellular tension. Nat. Rev. Mol. Cell Biol..

[34] Prevarskaya N., Skryma R., Shuba Y. (2011). Calcium in tumour metastasis: new roles for known actors. Nat. Rev. Cancer.

[35] Tsai F.-C., Kuo G.-H., Chang S.-W., Tsai P., Tsai F.-C., Kuo G.-H., Chang S.-W., Tsai P. (2015). Ca2+ signaling in cytoskeletal reorganization, cell migration, and cancer metastasis. Biomed. Res. Int..

[36] Bhatt A., Kaverina I., Otey C., Huttenlocher A. (2002). Regulation of focal complex composition and disassembly by the calcium-dependent protease calpain. J. Cell Sci..

[37] Wei C., Wang X., Zheng M., Cheng H. (2012). Calcium gradients underlying cell migration. Curr. Opin. Cell Biol..

[38] Tsai F.-C., Seki A., Yang H.W., Hayer A., Carrasco S., Malmersjö S., Meyer T. (2014). A polarized Ca2+, diacylglycerol and STIM1 signalling system regulates directed cell migration. Nat. Cell Biol..

[39] Tsai F.-C., Meyer T. (2012). Ca2+ pulses control local cycles of lamellipodia retraction and adhesion along the front of migrating cells. Curr. Biol..

[40] Wei C., Wang X., Chen M., Ouyang K., Song L.-S., Cheng H. (2009). Calcium flickers steer cell migration. Nature.

[41] Smyth J.T., Hwang S.Y., Tomita T., DeHaven W.I., Mercer J.C., Putney J.W. (2010). Activation and regulation of store-operated calcium entry. J. Cell Mol. Med..

[42] Stathopulos P.B., Ikura M. (2016). Store operated calcium entry: from concept to structural mechanisms. Cell Calcium.

[43] Yeung P.S.-W., Yamashita M., Prakriya M. (2016). Pore opening mechanism of CRAC channels. Cell Calcium.

[44] Schwindling C., Quintana A., Krause E., Hoth M. (2010). Mitochondria positioning controls local calcium influx in T cells. J. Immunol..

[45] Fonteriz J.A.R., Matesanz-Isabel J., Arias-del-Val Jessica J., Alvarez-Illera P., Montero M. (2016). Calcium entry pathways in non-excitable cells. Exp. Med. Biol..

[46] Desai S.P., Bhatia S.N., Toner M., Irimia D. (2013). Mitochondrial localization and the persistent migration of epithelial cancer cells. Biophys. J..

[47] Campello S., Lacalle R.A., Bettella M., Mañes S., Scorrano L., Viola A. (2006). Orchestration of lymphocyte chemotaxis by mitochondrial dynamics. J. Exp. Med..

[48] Zhao J., Zhang J., Yu M., Xie Y., Huang Y., Wolff D.W., Abel P.W., Tu Y. (2013). Mitochondrial dynamics regulates migration and invasion of breast cancer cells. Oncogene.

[49] Prudent J., Popgeorgiev N., Bonneau B., Thibaut J., Gadet R., Lopez J., Gonzalo P., Rimokh R., Manon S., Houart C., Herbomel P., Aouacheria A., Gillet G. (2013). Bcl-wav and the mitochondrial calcium uniporter drive gastrula morphogenesis in zebrafish. Nat. Commun..

[50] Webb S.E., Miller A.L. (2003). Calcium signalling during embryonic development. Nat. Rev. Mol. Cell Biol..

[51] Markova O., Lenne P.-F. (2012). Calcium signaling in developing embryos: focus on the regulation of cell shape changes and collective movements. Semin. Cell Dev. Biol..

[52] Xu S., Chisholm A.D. (2014). *C. elegans* Epidermal wounding induces a mitochondrial ROS burst that promotes wound repair. Dev. Cell.

[53] Ting S.B. (2005). A homolog of Drosophila grainy head is essential for epidermal integrity in mice. Science.

[54] Tran P.O.T., Hinman L.E., Unger G.M., Sammak P.J. (1999).

[55] Lansdown A.B.G., Path F.R.C. (2002). Perspective Article Calcium : a Potential Central Regulator in Wound Healing in the Skin.

[56] Pan X., Liu J., Nguyen T., Liu C., Sun J., Teng Y., Fergusson M.M., Rovira I.I., Allen M., Springer D.a., Aponte A.M., Gucek M., Balaban R.S., Murphy E., Finkel T. (2013). The physiological role of mitochondrial calcium revealed by mice lacking the mitochondrial calcium uniporter. Nat. Cell Biol..

[57] Wu Y., Rasmussen T.P., Koval O.M., a Joiner M.-L., Hall D.D., Chen B., Luczak E.D., Wang Q., Rokita A.G., Wehrens X.H.T., Song L.-S., Anderson M.E. (2015). The mitochondrial uniporter controls fight or flight heart rate increases. Nat. Commun..

[58] Murphy E., Pan X., Nguyen T., Liu J., Holmström K.M., Finkel T. (2014). Unresolved questions from the analysis of mice lacking MCU expression. Biochem. Biophys. Res. Commun..

[59] Prudent J., Popgeorgiev N., Bonneau B., Gillet G. (2015). Bcl-2 proteins, cell migration and embryonic development: lessons from zebrafish. Cell Death Dis..

[60] Bondarenko A.I., Jean-Quartier C., Parichatikanond W., Alam M.R., Waldeck-Weiermair M., Malli R., Graier W.F. (2013). Mitochondrial Ca(2+) uniporter (MCU)-dependent and MCU-independent Ca(2+) channels coexist in the inner mitochondrial membrane. Pflugers Arch..

[61] Antony A.N., Paillard M., Moffat C., Juskeviciute E., Correnti J., Bolon B., Rubin E., Csordás G., Seifert E.L., Hoek J.B., Hajnóczky G. (2016). MICU1 regulation of mitochondrial Ca(2+) uptake dictates survival and tissue regeneration. Nat. Commun..

[62] Liu J.C., Liu J., Holmstro K.M., Liu C., Murphy E., Menazza S., Parks R.J., Fergusson M.M., Yu Z. (2016). MICU1 Serves as a Molecular Gatekeeper to Prevent *in vivo* Mitochondrial Calcium Overload Article MICU1 Serves as a Molecular Gatekeeper to Prevent *in vivo* Mitochondrial Calcium Overload.

[63] Logan C.V., Szabadkai G., Sharpe J.a, Parry D.a, Torelli S., Childs A.-M., Kriek M., Phadke R., Johnson C.a, Roberts N.Y., Bonthron D.T., Pysden K.a, Whyte T., Munteanu I., Foley a.R., Wheway G., Szymanska K., Natarajan S., Abdelhamed Z.a, Morgan J.E., Roper H., Santen G.W.E., Niks E.H., van der Pol W.L., Lindhout D., Raffaello A., De Stefani D., den Dunnen J.T., Sun Y., Ginjaar I., Sewry C.a, Hurles M., Rizzuto R., Duchen M.R., Muntoni F., Sheridan E. (2013). Loss-of-function mutations in MICU1 cause a brain and muscle disorder linked to primary alterations in mitochondrial calcium signaling. Nat. Genet..

[64] Lewis-Smith D., Kamer K.J., Griffin H., Childs A.-M., Pysden K., Titov D., Duff J., Pyle A., Taylor R.W., Yu-Wai-Man P., Ramesh V., Horvath R., Mootha V.K., Chinnery P.F. (2016). Homozygous deletion in MICU1 presenting with fatigue and lethargy in childhood. Neurol. Genet..

[65] Bhosale G., Sharpe J.A., Koh A., Kouli A., Szabadkai G., Duchen M.R. (2017). Pathological consequences of MICU1 mutations on mitochondrial calcium signalling and bioenergetics ☆. BBA - Mol. Cell Res..

[66] Marchi S., Lupini L., Patergnani S., Rimessi A., Missiroli S., Bonora M., Giorgi C., Bononi A., Corra F., De Marchi E., Poletti F., Gafa R., Lanza G., Negrini M., Rizzuto R. (2013).

[67] Tang S., Wang X., Shen Q., Yang X., Yu C., Cai C., Cai G., Meng X., Zou F. (2015). Mitochondrial Ca(2+) uniporter is critical for store-operated Ca(2+) entry-dependent breast cancer cell migration. Biochem. Biophys. Res. Commun..

[68] Hall D.D., Wu Y., Domann F.E., Spitz D.R., Anderson M.E. (2014). Mitochondrial calcium uniporter activity is dispensable for MDA-MB-231 breast carcinoma cell survival. PLoS One.

[69] Tosatto A., Sommaggio R., Kummerow C., Bentham R.B., Blacker T.S., Berecz T., Duchen M.R., Rosato A., Bogeski I., Szabadkai G., Rizzuto R., Mammucari C. (2016). The mitochondrial calcium uniporter regulates breast cancer progression via HIF-1. EMBO Mol. Med..

[70] Prudent J., Popgeorgiev N., Gadet R., Deygas M., Rimokh R., Gillet G. (2016). Mitochondrial Ca2+ uptake controls actin cytoskeleton dynamics during cell migration. Sci. Rep..

[71] Pendin D., Greotti E., Pozzan T. (2014). The elusive importance of being a mitochondrial Ca2+ uniporter. Cell Calcium.

[72] Hoffman N.E.E., Chandramoorthy H.C.C., Shamugapriya S., Zhang X., Rajan S., Mallilankaraman K., Gandhirajan R.K.K., Vagnozzi R.J.J., Ferrer L.M.M., Sreekrishnanilayam K., Natarajaseenivasan K., Vallem S., Force T., Choi E.T.T., Cheung J.Y.Y., Madesh M. (2013). MICU1 motifs Define mitochondrial calcium uniporter binding and activity. Cell Rep..

[73] Quan X., Nguyen T.T., Choi S.-K., Xu S., Das R., Cha S.-K., Kim N., Han J., Wiederkehr A., Wollheim C.B., Park K.-S. (2015). Essential role of mitochondrial Ca2+ uniporter in the generation of mitochondrial pH gradient and metabolism-secretion coupling in insulin-releasing cells. J. Biol. Chem..

[74] Drago I., De Stefani D., Rizzuto R., Pozzan T. (2012). Mitochondrial Ca^2+^ Uptake Contributes to Buffering Cytoplasmic Ca^2+^Peaks in Cardiomyocytes. 10.1073/pnas.1210718109/-/DCSupplemental.www.pnas.org/cgi/doi/10.1073/pnas.1210718109.

[75] Tarasov A.I., Semplici F., Ravier M.a, Bellomo E.a, Pullen T.J., Gilon P., Sekler I., Rizzuto R., Rutter G.a (2012). The mitochondrial Ca(2+) uniporter MCU is essential for glucose-induced ATP increases in pancreatic β-cells. PLoS One.

[76] Tarasov A.I., Semplici F., Li D., Rizzuto R., Ravier M.A., Gilon P., Rutter G.A. (2013). Frequency-dependent mitochondrial Ca2+ accumulation regulates ATP synthesis in pancreatic cells. Pflugers Arch. Eur. J. Physiol..

[77] Fouqué A., Lepvrier E., Debure L., Gouriou Y., Malleter M., Delcroix V., Ovize M., Ducret T., Li C., Hammadi M., Vacher P., Legembre P. (2016). The apoptotic members CD95, BclxL, and Bcl-2 cooperate to promote cell migration by inducing Ca2+ flux from the endoplasmic reticulum to mitochondria. Cell Death Differ..

[78] Mallilankaraman K., Doonan P., Cárdenas C., Chandramoorthy H.C., Müller M., Miller R., Hoffman N.E., Gandhirajan R.K., Molgó J., Birnbaum M.J., Rothberg B.S., Mak D.O.D., Foskett J.K., Madesh M. (2012). MICU1 is an essential gatekeeper for mcu-mediated mitochondrial Ca 2+ uptake that regulates cell survival. Cell.

[79] Ishikawa K., Takenaga K., Akimoto M., Koshikawa N., Yamaguchi A., Imanishi H., Nakada K., Honma Y., Hayashi J. (2008). ROS-generating mitochondrial DNA mutations can regulate Tumor cell metastasis. Science.

[80] Brookes P.S., Yoon Y., Robotham J.L., Anders M.W., Sheu S. (2004). Calcium, ATP, and ROS : a Mitochondrial Love-hate Triangle.

[81] Chipuk J.E., Moldoveanu T., Llambi F., Parsons M.J., Green D.R. (2010). The BCL-2 family reunion. Mol. Cell.

[82] Ke H., Parron V.I., Reece J., Zhang J.Y., Akiyama S.K., French J.E. (2010). BCL2 inhibits cell adhesion, spreading, and motility by enhancing actin polymerization. Cell Res..

[83] Koehler B.C., Scherr A.-L., Lorenz S., Urbanik T., Kautz N., Elssner C., Welte S., Bermejo J.L., Jäger D., Schulze-Bergkamen H. (2013). Beyond cell death - antiapoptotic Bcl-2 proteins regulate migration and invasion of colorectal cancer cells *in vitro*. PLoS One.

[84] Bonneau B., Prudent J., Popgeorgiev N., Gillet G. (2013). Non-apoptotic roles of Bcl-2 family: the calcium connection. Biochim. Biophys. Acta.

[85] Huang H., Hu X., Eno C.O., Zhao G., Li C., White C. (2013). An interaction between Bcl-xL and the voltage-dependent anion channel (VDAC) promotes mitochondrial Ca2+ uptake. J. Biol. Chem..

[86] Monaco G., Decrock E., Arbel N., Van Vliet A.R., La Rovere R.M., De Smedt H., Parys J.B., Agostinis P., Leybaert L., Shoshan-Barmatz V., Bultynck G. (2015). The BH4 domain of anti-apoptotic Bcl-XL, but not that of the related Bcl-2, limits the voltage-dependent anion channel 1 (VDAC1)-mediated transfer of pro-apoptotic Ca^2+^ signals to mitochondria. J. Biol. Chem..

[87] Huang H., Shah K., Bradbury N.a, Li C., White C. (2014). Mcl-1 promotes lung cancer cell migration by directly interacting with VDAC to increase mitochondrial Ca(2+) uptake and reactive oxygen species generation. Cell Death Dis..

[88] Choi S., Chen Z., Tang L.H., Fang Y., Shin S.J., Panarelli N.C., Chen Y.-T., Li Y., Jiang X., Du Y.-C.N. (2016). Bcl-xL promotes metastasis independent of its anti-apoptotic activity. Nat. Commun..

[89] Sundaresan M F.T., Yu Z.X., Ferrans V.J., Irani K. (1995). Requirement for generation of H2O2 for Platelet-derived growth factor signal transduxtion. Science.

[90] Hurd T.R., DeGennaro M., Lehmann R. (2012). Redox regulation of cell migration and adhesion. Trends Cell Biol..

[91] Choi M.H., Lee I.K., Kim G.W., Kim B.U., Han Y.-H., Yu D.-Y., Park H.S., Kim K.Y., Lee J.S., Choi C., Bae Y.S., Lee B.I., Rhee S.G., Kang S.W. (2005). Regulation of PDGF signalling and vascular remodelling by peroxiredoxin II. Nature.

[92] Levy J.R., Holzbaur E.L.F., Parsons J.T., Horwitz A.R., Schwartz M.a, Larson D.R., Singer R.H., Zenklusen D., Campellone K.G., Welch M.D., Schneider-Poetsch T., Ju J., Eyler D.E., Dang Y., Bhat S., Merrick W.C., Green R., Shen B., Liu J.O., Schnitzer J., Storm N. (2010). Cell adhesion: integrating cytoskeletal dynamics and cellular tension. Mol. Cell.

[93] Nimnual A.S., Taylor L.J., Bar-Sagi D. (2003). Redox-dependent downregulation of Rho by Rac. Nat. Cell Biol..

[94] Xu S., Chisholm A.D. (2011). A Gαq-Ca2+ signaling pathway promotes actin-mediated epidermal wound closure in *c. elegans*. Curr. Biol..

[95] Xu S., Chisholm A.D. (2014). Article *c. elegans* epidermal wounding induces a mitochondrial ROS burst that promotes wound repair. Dev. Cell.

[96] Booth D.M., Enyedi B., Geiszt M., Várnai P., Hajnóczky G. (2016). Redox nanodomains are induced by and control calcium signaling at the ER-mitochondrial interface. Mol. Cell.

[97] Cunniff B., Mckenzie A.J., Heintz N.H., Howe A.K. (2016). AMPK activity regulates trafficking of mitochondria to the leading edge during cell migration and matrix invasion.

[98] Hoth M., Fanger C.M., Lewis R.S. (1997).

[99] Gilabert J.A., Parekh A.B. (2000).

[100] Malli R., Frieden M., Osibow K., Zoratti C., Mayer M., Demaurex N., Graier W.F. (2003). Sustained Ca2+ transfer across mitochondria is essential for mitochondrial Ca2+ buffering, store-operated Ca2+ entry, and Ca2+ store refilling. J. Biol. Chem..

[101] Glitsch M.D., Bakowski D., Parekh A.B. (2002). Store-operated Ca^2+^ entry depends on mitochondrial Ca^2+^ uptake. EMBO J..

[102] Naghdi S., Waldeck-Weiermair M., Fertschai I., Poteser M., Graier W.F., Malli R. (2010). Mitochondrial Ca2+ uptake and not mitochondrial motility is required for STIM1-Orai1-dependent store-operated Ca2+ entry. J. Cell Sci..

[103] Demaurex N., Poburko D., Frieden M. (2009). Regulation of plasma membrane calcium fluxes by mitochondria. Biochim. Biophys. Acta.

[104] Quintana A., Schwarz E.C., Schwindling C., Lipp P., Kaestner L., Hoth M. (2006). Sustained activity of calcium release-activated calcium channels requires translocation of mitochondria to the plasma membrane. J. Biol. Chem..

[105] Quintana A., Schwindling C., Wenning A.S., Becherer U., Rettig J., Schwarz E.C., Hoth M. (2007). T cell activation requires mitochondrial translocation to the immunological synapse. Proc. Natl. Acad. Sci. U. S. A..

[106] Contento R.L., Campello S., Trovato A.E., Magrini E., Anselmi F., Viola A. (2010). Adhesion shapes T cells for prompt and sustained T-cell receptor signalling. EMBO J..

[107] Samanta K., Douglas S., Parekh A.B. (2014).

[108] González-Sánchez P., Pla-Martín D., Martínez-Valero P., Rueda C.B., Calpena E., del Arco A., Palau F., Satrústegui J. (2017). CMT-linked loss-of-function mutations in GDAP1 impair store-operated Ca2+ entry-stimulated respiration. Sci. Rep..

[109] Deak A.T., Blass S., Khan M.J., Groschner L.N., Waldeck-Weiermair M., Hallström S., Graier W.F., Malli R. (2014). IP3-mediated STIM1 oligomerization requires intact mitochondrial Ca2+ uptake. J. Cell Sci..

[110] Chen Y.-T., Chen Y.-F., Chiu W.-T., Wang Y.-K., Chang H.-C., Shen M.-R. (2013). The ER Ca^2+^ sensor STIM1 regulates actomyosin contractility of migratory cells. J. Cell Sci..

[111] Yang S., Zhang J.J., Huang X.Y. (2009). Orai1 and STIM1 Are Critical for Breast Tumor Cell Migration and Metastasis. Cancer Cell.

[112] Korzeniowski M.K., Szanda G., Balla T., Spät A. (2009). Store-operated Ca2+ influx and subplasmalemmal mitochondria. Cell Calcium.

[113] Giacomello M., Drago I., Bortolozzi M., Scorzeto M., Gianelle A., Pizzo P., Pozzan T. (2010). Ca2+ Hot Spots on the Mitochondrial Surface Are Generated by Ca2+ Mobilization from Stores, but Not by Activation of Store-Operated Ca2+ Channels. Mol. Cell.

[114] Schwarz T.L. (2013). Mitochondrial trafficking in neurons. Cold Spring Harb. Perspect. Biol. Biol..

[115] Wang X., Schwarz T.L. (2009). The mechanism of Ca2+ -dependent regulation of kinesin-mediated mitochondrial motility. Cell.

[116] Macaskill A.F., Rinholm J.E., Twelvetrees A.E., Arancibia-Carcamo I.L., Muir J., Fransson A., Aspenstrom P., Attwell D., Kittler J.T. (2009). Article Miro1 is a calcium sensor for glutamate receptor-dependent localization of mitochondria at synapses. Neuron.

[117] Chang K.T., Niescier R.F., Min K. (2011). Mitochondrial matrix Ca^2+^ as an intrinsic signal regulating mitochondrial motility in axons. 10.1073/pnas.1106862108/-/DCSupplemental.www.pnas.org/cgi/doi/10.1073/pnas.1106862108.

[118] Ferreira-da-silva A., Valacca C., Rios E., Pópulo H. (2015). Mitochondrial Dynamics Protein Drp1 Is Overexpressed in Oncocytic Thyroid Tumors and Regulates Cancer Cell Migration.

[119] Kashatus J.A., Nascimento A., Myers L.J., Sher A., Byrne F.L., Hoehn K.L., Counter C.M., Kashatus D.F. (2015). Erk2 phosphorylation of Drp1 promotes mitochondrial fission and MAPK-driven tumor growth. Mol. Cell.

[120] Serasinghe M.N., Wieder S.Y., Renault T.T., Elkholi R., Asciolla J.J., Yao J.L., Jabado O., Hoehn K., Kageyama Y., Sesaki H., Chipuk J.E. (2015). Mitochondrial division is requisite to RAS-induced transformation and targeted by oncogenic MAPK pathway inhibitors. Mol. Cell.

[121] Jung J.-U., Ravi S., Lee D.W., McFadden K., Kamradt M.L., Toussaint L.G., Sitcheran R. (2016). NIK/MAP3K14 regulates mitochondrial dynamics and trafficking to promote cell invasion. Curr. Biol..

[122] Cereghetti G.M., Stangherlin a, Martins de Brito O., Chang C.R., Blackstone C., Bernardi P., Scorrano L. (2008). Dephosphorylation by calcineurin regulates translocation of Drp1 to mitochondria. Proc. Natl. Acad. Sci. U. S. A..

[123] Cribbs J.T., Strack S. (2007). Reversible phosphorylation of Drp1 by cyclic AMP-dependent protein kinase and calcineurin regulates mitochondrial fission and cell death. EMBO Rep..

[124] Hom J.R., Gewandter J.S., Michael L., Sheu S., Yoon Y. (2007). Thapsigargin Induces Biphasic Fragmentation of Mitochondria through Calcium-mediated Mitochondrial Fission and Apoptosis.

[125] Zhao L., Li S., Wang S., Yu N., Liu J. (2015). Biochemical and Biophysical Research Communications The effect of mitochondrial calcium uniporter on mitochondrial fi ssion in hippocampus cells ischemia/reperfusion injury. Biochem. Biophys. Res. Commun..

[126] Liang N., Wang P., Wang S., Li S., Li Y., Wang J., Wang M. (2014). Role of mitochondrial calcium uniporter in regulating mitochondrial fission in the cerebral cortexes of living rats. J. Neural Transm..

[127] Zheng X., Chen M., Meng X., Chu X., Cai C., Zou F. (2017). Phosphorylation of dynamin-related protein 1 at Ser616 regulates mitochondrial fission and is involved in mitochondrial calcium uniporter-mediated neutrophil polarization and chemotaxis. Mol. Immunol..

[128] Kim B., Takeuchi A., Hikida M., Matsuoka S. (2016). Roles of the mitochondrial Na+-Ca2+ exchanger, NCLX, in B lymphocyte chemotaxis. Sci. Rep..

[129] Tang S., Wang X., Shen Q., Yang X., Yu C., Cai C., Cai G., Meng X., Zou F. (2015). Biochemical and Biophysical Research Communications Mitochondrial Ca 2 þ uniporter is critical for store-operated Ca 2 þ entry-dependent breast cancer cell migration. Biochem. Biophys. Res. Commun..

[130] Du Y.C.N., Lewis B.C., Hanahan D., Varmus H. (2007). Assessing tumor progression factors by somatic gene transfer into a mouse model: Bcl-xL promotes islet tumor cell invasion. PLoS Biol..

